# Availability of Medical and Recreational Marijuana Stores and Neighborhood Characteristics in Colorado

**DOI:** 10.1155/2016/7193740

**Published:** 2016-04-24

**Authors:** Yuyan Shi, Kristin Meseck, Marta M. Jankowska

**Affiliations:** ^1^Department of Family Medicine and Public Health, University of California, 9500 Gilman Drive, La Jolla, CA 92093-0622, USA; ^2^California Institute for Telecommunications & Information Technology, University of California, La Jolla, CA 92093, USA

## Abstract

*Objective*. To examine the availability of marijuana stores in Colorado and associations with neighborhood characteristics.* Methods*. The addresses for 650 medical and recreational marijuana stores were geocoded and linked to the characteristics of 1249 census tracts in Colorado. Accounting for spatial autocorrelations, autologistic regressions were used to quantify the associations of census tract socioeconomic characteristics with the availability of marijuana stores.* Results*. Regardless of store types, marijuana stores were more likely to locate in neighborhoods that had a lower proportion of young people, had a higher proportion of racial and ethnic minority population, had a lower household income, had a higher crime rate, or had a greater density of on-premise alcohol outlets. The availability of medical and recreational marijuana stores was differentially correlated with household income and racial and ethnic composition.* Conclusions*. Neighborhood disparities existed in the availability of marijuana stores, and associations between availability of stores and neighborhood characteristics varied by store types. This study highlighted the need for regulatory measures to prevent marijuana related outcomes in high risk neighborhoods.

## 1. Introduction

In the USA, the prevalence of marijuana use is high among both adolescents and adults. In 2013, an estimated 19.8 million people older than 12 years were past-month marijuana users, accounting for 7.5% of the population in the age group [1]. Despite a declining trend observed in cigarette smoking and alcohol drinking in the past few years, marijuana use has remained stable [2].

Marijuana use, as other substance use behaviors, is influenced by neighborhood physical, economic, and social environmental factors. Since 1996, 23 states and Washington DC have adopted policies to legalize marijuana for medical use. Recently, recreational marijuana use was also legalized in 4 states and Washington DC. These legal and policy changes have dramatically changed the neighborhood environments for marijuana use. One of the most notable changes is the emergence of marijuana stores, which provide legal access to marijuana and potentially modify social norms related to marijuana use within the neighborhood. Initial evidence has suggested the associations between the availability of medical marijuana stores and higher rates of marijuana use and abuse in California cities [3, 4].

The prevalence of marijuana use and abuse is not homogeneous. Demographically and socioeconomically vulnerable populations are at higher risks of using or abusing marijuana. For example, people who are younger, racial and ethnic minorities, having low income, less educated, and living in urban and disordered areas had higher proportions of marijuana use and marijuana use disorders [5, 6]. If demographically and socioeconomically vulnerable neighborhoods had a greater availability of marijuana stores, the disparity in marijuana store distributions may lead to differential exposures to marijuana and exacerbate marijuana use and related outcomes in demographically or socioeconomically vulnerable populations.

The availability of marijuana stores around schools is also of public health concern. As suggested by literature on tobacco and alcohol [7–10], close proximity of marijuana stores to schools may increase the risks of marijuana use among adolescents who are at a particularly high risk of developing marijuana use disorders and other negative health consequences [11]. Currently, Colorado has zoning regulations that keep marijuana stores 1,000 feet from schools. However, stores may still locate within walking distance beyond the 1,000-feet limit. It remains unknown to what extent stores locate within walkable distance that adolescents can easily reach. If some schools have stores available within walking distance while other schools do not, the differential exposures to marijuana may lead to disparities in marijuana use prevalence among adolescents.

The associations between the availability of medical marijuana stores and neighborhood characteristics have been evaluated in major cities in California and Colorado. Evidence from California suggested that medical marijuana stores were more likely to be located in areas with higher proportion of Hispanic residents and higher density of alcohol outlets and higher rates of poverty and primarily zoned as commercial [4, 12, 13]. Although the availability of medical marijuana stores in Denver, Colorado, did not differ by neighborhood minority composition or poverty status, it was associated with higher crime rates [14]. There have not been any studies focused on the distribution of recreational marijuana stores partly because recreational marijuana was not legalized until 2014. Theoretically, medical marijuana and recreational marijuana stores target different segments of the marijuana user population, and two types of stores could be differentially available in various neighborhoods.

Colorado was among the first few states to implement medical marijuana policies and the first state to implement recreational marijuana policies in the USA. Colorado State and local jurisdictions have implemented various policies to regulate the distribution of marijuana stores, but little is known about whether marijuana stores are disproportionately located in the entire state of Colorado as a result and to what extent different types of marijuana stores are available in different neighborhoods. This study provided the first statewide evaluation on the availability of both medical marijuana stores and recreational marijuana stores and their associations with neighborhood characteristics on demographics, socioeconomic status, crime, and alcohol outlets in Colorado. It also assessed marijuana store locations by distance to schools. Medical marijuana stores and recreational marijuana stores were evaluated separately. This study was expected to illuminate policy implications to public health policy makers and urban planners regarding zoning, density control, and licensing regulations on marijuana stores and prevention program targeted towards neighborhoods at high risks of clustering marijuana users.

## 2. Method

A cross-sectional ecological study was conducted to examine the relationships between the availability of marijuana stores and neighborhood characteristics. Neighborhood was defined as census tract in this study, and all census tracts in Colorado were used in the analysis (*N* = 1,249). Census tracts in the USA are small statistical subdivisions that typically coincide within the boundaries of administrative areas such as cities or towns. The population size of a census tract generally ranges between 1200 and 8000 and population characteristics and socioeconomic status within a census tract are relatively homogeneous [15]. The study was conducted in 2015.

### 2.1. Availability of Marijuana Stores

The outcome variables included binary indicators to represent whether or not a census tract had any marijuana stores. Measures were separately created for the availability of (1) any type of marijuana store, (2) medical marijuana store, and (3) recreational marijuana store. We obtained directories of licensed marijuana stores from the Enforcement Division, Colorado Department of Revenue, which provided detailed physical addresses for all medical and recreational marijuana stores as of August, 2015. A total of 650 licensed marijuana stores were identified and their point locations were geocoded and mapped using ArcMap 10.2. Among the 650 stores, 270 were only registered as medical marijuana stores and 139 were only registered as recreational marijuana stores with 241 stores registered as both medical and recreational marijuana stores.

### 2.2. Neighborhood Characteristics

Data for census tract characteristics were obtained from multiple sources. The following variables were derived from US Census, American Community Survey, and Public Elementary and Secondary School Universe Survey: (1) population size, (2) land area, (3) proportion of population under age of 21 (people who have no legal access to marijuana stores in Colorado), (4) proportion of racial and ethnic minority population (race and ethnicity other than Non-Hispanic White), (5) median household annual income, (6) unemployment rate, and (7) density of primary and secondary schools per square mile.

We used the crime risk index developed by ESRI based on FBI Uniform Crime Report databases. The index represented standardized number of crime cases in murder, rape, robbery, assault, burglary, theft, and motor vehicle theft categories, with continuous values ranging between 6 and 739 for Colorado census tracts. Detailed methodology for the development of the crime risk index can be found elsewhere [16]. For easy interpretation, continuous crime risk index was converted into three tertiles to represent low, medium, and high crime rate in the neighborhoods.

Directories of licensed alcohol retail outlets were obtained from the Enforcement Division, Colorado Department of Revenue, in 2015. Two continuous variables were created, respectively, to represent the density of on-premise alcohol outlets and off-premise alcohol outlets per square mile.

### 2.3. Analysis

The cross-sectional examination of correlations was conducted at the census tract level. We compared census tract characteristics by the availability of marijuana stores and conducted* t*-test to test between-group differences. We plotted the counts of marijuana stores by store types. We created buffers around schools (0–1000 feet, 1000 feet–1 mile, and above 1 mile) to examine the distribution of marijuana stores within each buffer. Because census tracts are “autocorrelated” in the sense that nearby units share more similarities relative to distant ones, we used Global Moran's* I* statistics to detect spatial autocorrelation between census tracts. We found some spatial autocorrelation between the census tracts in terms of the availability of marijuana stores (Moran's* I* = 0.099, *p* < 0.001). We therefore assessed the associations between the availability of marijuana stores and neighborhood characteristics by multivariate autologistic regressions to account for spatial autocorrelation [17]. The outcome variables for the availability of any type of marijuana stores, medical marijuana stores, and recreational marijuana stores were evaluated, respectively. The autologistic analyses were estimated using R packages [18].* p* values smaller than 0.05 were considered statistically significant.

## 3. Results

### 3.1. Descriptive Statistics

**Figure 1:**
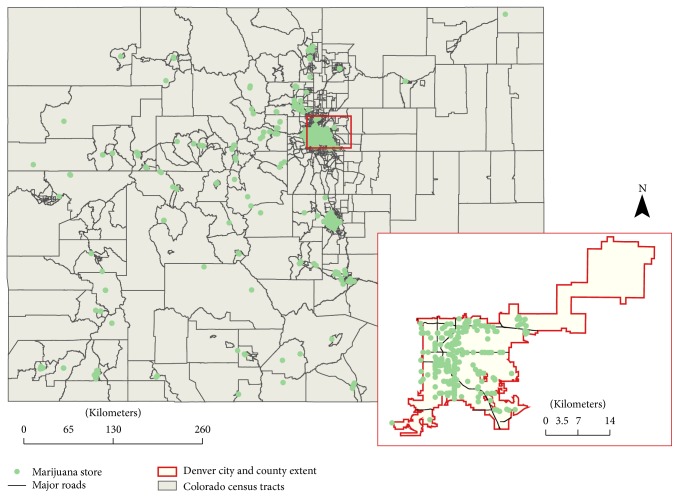
Spatial distribution of marijuana stores at census tract level in Colorado (census tract *N* = 1,249).

**Figure 2:**
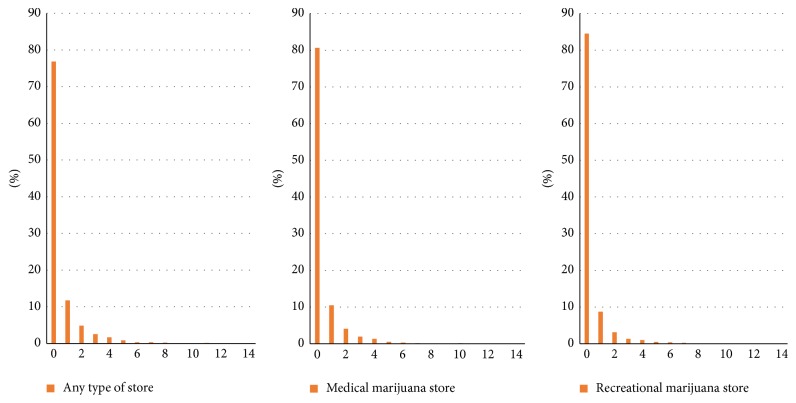
Density of marijuana stores at census tract level in Colorado (census tract *N* = 1,249).

Figure 1 illustrated the geographic distribution of marijuana stores in Colorado regardless of store types.  Figure 2 plotted the statistical distribution of the count of marijuana stores. Out of 1,249 census tracts, 289 or 23.14% had at least one marijuana store available regardless of store types; 242 census tracts had at least one medical marijuana store; and 193 census tracts had at least one recreational marijuana store. Within census tracts that have at least one store, the average numbers of stores of any type, medical marijuana stores, and recreational marijuana stores were 2.25, 2.11, and 1.99, respectively.

Table 1 reported summary statistics for census tract characteristics by the availability of marijuana stores. On average, the population size was 4030 with 28.11% population 21 years of age or younger. Racial and ethnic minorities accounted for 29.74% of the total population. The median household income was $61,943.81, and the unemployment rate was 6.69%. In terms of crime, the standardized crime index was 100.84 on average for all census tracts. The density for schools and on-premise and off-premise alcohol outlets were 1.20, 4.47, and 1.82 per square mile, respectively.

All neighborhood characteristics but population size and land area differed significantly between census tracts with and without any type of marijuana stores (Table 1). Specifically, the census tracts with any type of marijuana stores had a lower percentage of young population, larger proportion of racial and ethnic minorities, lower medium household income, larger unemployment rate, and higher crime rate. The school and alcohol outlets densities were also higher in census tracts with any type of marijuana stores compared to census tracts without any stores.

Table 2 reported the availability of marijuana stores around primary and secondary schools in Colorado. Within 1000-feet radius, 1.72% schools had at least one marijuana store regardless of type; within 1000 feet–1 mile, 32.73% schools had at least one marijuana store regardless of type. The distance to the nearest marijuana stores for the remaining 65.55% schools was greater than 1 mile. A slightly greater proportion of medical marijuana stores were located within 1-mile buffer of schools (28.66%) relative to recreational marijuana stores (24.56%).

### 3.2. Regression Analysis

The results from autologistic regression models were reported in Table 3. Regardless of store types, marijuana stores were more likely to locate in census tracts that had a larger population size (*p* < 0.001), larger land area (*p* = 0.006), lower proportion of population under age of 21 (*p* = 0.010), larger proportion of racial and ethnic minorities (*p* = 0.039), and lower household income (*p* = 0.044). Census tracts with medium and high crime indices were 2.84 (*p* < 0.001) and 3.48 (*p* < 0.001) times as likely to have marijuana stores as census tracts with low crime index. Unemployment rate and density of schools were found to be unrelated to the availability of marijuana stores.

We observed heterogeneities in the correlations between census tract characteristics and the availability of marijuana stores by store types. For example, the census tracts that had a high proportion of racial and ethnicity minorities were more likely to have recreational marijuana store available (*p* < 0.001) but not more likely to have medical marijuana stores. A lower household income was associated with a greater likelihood of having medical marijuana stores (*p* = 0.022), but its relationship with the availability of recreational marijuana stores was not significant.

## 4. Discussion

Based on the statewide analysis of marijuana stores in Colorado, this study found that the availability of marijuana stores was associated with certain neighborhood characteristics. Consistent with the observations in Denver, Colorado, marijuana stores throughout the Colorado State were more likely to locate in neighborhoods with high crime rates [14]. Our findings also supported the evidence from California major cities which demonstrated greater availability of medical marijuana stores in neighborhoods that had higher proportion of minority, higher level of poverty, and higher density of alcohol outlets [4, 12, 13].

No empirical studies have explored the mechanisms behind the observed correlations between the availability of marijuana stores and neighborhood characteristics. The disproportionally high concentration of marijuana stores in areas with high crime rates and alcohol outlet density could be a result of retail concentrations, which provide land and staffing for commercial use but also increase chances for crime cases [14]. The clustering of marijuana stores in areas with lower income households and higher proportion of minorities might be explained by the high prevalence of marijuana use and favorable social norms associated with marijuana use in the population. Such neighborhoods could provide a potentially large pool of clients as well as a supportive environment for the store establishments. Future research is much needed to uncover the causal relationships of these associations.

The availability of marijuana stores may have impacts on the disparities in marijuana use and related health consequences. People who are at a higher risk of marijuana use, such as younger age groups, may reduce use due to a smaller exposure to marijuana stores in neighborhoods that have a high proportion of young population. In contrast, marijuana use may be increased in racial and ethnic minority population and population with low income and high crime rates because the neighborhoods clustered with these types of vulnerable population had a greater availability of marijuana stores. Some literature has shown that alcohol may be a gateway substance to the use of marijuana; therefore the higher likelihood of marijuana stores locating in neighborhoods with higher densities of alcohol outlets could have implications for uptake of marijuana [19]. Because of the concern about the negative impacts of marijuana stores, Denver recently proposed extending the moratorium on new recreational marijuana stores and banning new licenses for medical marijuana stores [20]. Empirical research that evaluates the consequences of marijuana store availability at neighbored level is warranted to inform public health and urban planning policy makers.

This study revealed differential availabilities of marijuana stores by store types. The availability of medical marijuana stores and recreational marijuana stores demonstrated different associations with racial and ethnic composition and household income. The different distribution of medical and recreational marijuana stores in the neighborhoods highlighted the need for public health policy makers and urban planners to consider store type upon developing regulations on stores.

The availability of marijuana stores around primary and secondary schools is of particular concern. Although very few marijuana stores violated the 1000-feet zone regulation, around a third of schools in Colorado had at least one marijuana store available within walkable distance (1 mile) to school boundaries. Even though students under age of 21 are not legally allowed to purchase marijuana, they may still gain access if age restriction in marijuana stores is not strictly enforced. In addition, the advertisements for marijuana in the neighborhoods may modify the social norms related to marijuana among adolescents and young adults.

Several limitations of this study are noteworthy. First, this study was a cross-sectional analysis that may be subject to selection bias and unobserved heterogeneities. The associations should not be interpreted as causal inferences. Second, although the census tract is often used as a proxy for neighborhood, its characteristics may not represent the real neighborhood in which marijuana stores are located and individuals are living and working, particularly in large rural census tracts. Third, the reported associations did not account for spatial correlations between two types of marijuana stores. Fourth, the binary outcome of presence or absence of marijuana stores likely obscures some of the clustering effects of multiple stores found in urban areas. Last, the study findings may not be generalized to states other than Colorado.

## 5. Conclusion

This study suggested that a few unfavorable neighborhood measures were associated with a greater availability of marijuana stores, and different types of marijuana stores were differentially available in the neighborhoods. The findings underscored the need for regulatory measures to reduce the disparities in marijuana store locations with particular attention to the differences between store types. Considering that differential access to marijuana may influence individuals' marijuana use decisions and purchase behaviors, prevention programs that target high risk neighborhoods are also recommended.

## Figures and Tables

**Table 1:** Neighborhood characteristics by type of marijuana stores in Colorado (census tract *N* = 1,249).

Census tract characteristics	All census tracts	Census tracts without any marijuana stores	Census tracts with marijuana stores			Test for difference between tracts with and without any type of marijuana stores (*p* value)
			Any type of marijuana stores	Medical marijuana stores	Recreational marijuana stores	
Number of census tracts, *N* (%)	1,249 (100)	960 (76.86)	289 (23.14)	242 (19.38)	193 (15.45)	
Population size, thousand	4.03	3.99	4.19	4.18	4.11	0.10
Land area, hundred square miles	0.83	0.82	0.87	0.64	1.08	0.81
Proportion of population age <21	0.28	0.28	0.25	0.25	0.25	<0.001^*∗∗∗*^
Proportion of racial and ethnic minority (not Non-Hispanic White)	0.30	0.28	0.35	0.34	0.36	<0.001^*∗∗∗*^
Median household income, tens of thousands $	6.19	6.49	5.18	5.18	5.31	<0.001^*∗∗∗*^
Unemployment rate	0.067	0.065	0.073	0.072	0.069	0.0026^*∗∗*^
Schools, density/sq mile	1.20	1.12	1.49	1.61	1.57	0.0011^*∗∗*^
Crime index	100.84	92.57	128.33	130.71	128.20	<0.001^*∗∗∗*^
Alcohol on-premise outlets, density/sq mile	4.47	2.76	10.16	11.32	12.57	<0.001^*∗∗∗*^
Alcohol off-premise outlets, density/sq mile	1.82	1.46	3.04	3.20	2.92	<0.001^*∗∗∗*^

^*∗∗∗*^ *p* < 0.001 and ^*∗∗*^ *p* < 0.1.

**Table 2:** Availability of marijuana stores within various buffers around schools in Colorado (schools *N* = 2,215).

Radius of buffers around schools	Number of schools with any type of marijuana stores in the buffer *N* (%)	Number of schools with medical marijuana stores in the buffer *N* (%)	Number of schools with recreational marijuana stores in the buffer *N* (%)
0–1000 feet	38 (1.72)	30 (1.35)	25 (1.13)
1000 feet–1 mile	725 (32.73)	605 (27.31)	519 (23.43)
Above 1 mile	1452 (65.55)	1580 (71.33)	1671 (75.44)

**Table 3:** Autologistic regression: availability of marijuana stores in Colorado and neighborhood characteristics.

	Availability of marijuana stores		
	Odds ratio (standard error)		
	Any type of marijuana stores	Medical marijuana stores	Recreational marijuana stores
Population size, thousand	1.19 (0.051)^*∗∗∗*^	1.19 (0.056)^*∗∗∗*^	1.13 (0.055)^*∗∗*^
Land area, hundred square miles	1.06 (0.022)^*∗∗*^	1.02 (0.029)	1.09 (0.026)^*∗∗∗*^
Proportion of population age <21	0.014 (0.024)^*∗∗*^	0.017 (0.032)^*∗*^	0.0056 (0.013)^*∗*^
Proportion of racial and ethnic minority	3.12 (1.72)^*∗*^	1.63 (0.97)	17.50 (12.57)^*∗∗∗*^
Median household income, tens of thousands $	0.91 (0.040)^*∗*^	0.89 (0.041)^*∗*^	1.02 (0.049)
Unemployment rate	0.74 (1.55)	0.61 (1.36)	0.039 (0.10)
Schools, density/sq mile	0.94 (0.040)	0.99 (0.042)	0.97 (0.048)
Crime index			
Low (reference)	1	1	1
Medium	2.84 (0.63)^*∗∗∗*^	2.75 (0.67)^*∗∗∗*^	3.47 (0.96)^*∗∗∗*^
High	3.48 (0.84)^*∗∗∗*^	3.34 (0.88)^*∗∗∗*^	3.47 (1.07)^*∗∗∗*^
Alcohol on-premise outlets, density/sq mile	1.02 (0.013)^*∗*^	1.02 (0.013)^*∗*^	1.04 (0.015)^*∗∗*^
Alcohol off-premise outlets, density/sq mile	1.06 (0.036)	1.06 (0.036)	0.95 (0.033)

^*∗∗∗*^ *p* < 0.001, ^*∗∗*^ *p* < 0.1, and ^*∗*^ *p* < 0.05.
